# Examining Explicit Stereotype Perceptions of Colorectal Cancer Screening and Diagnosis in the Hispanic Community

**DOI:** 10.1007/s13187-025-02609-y

**Published:** 2025-03-26

**Authors:** Aidan Foley, Bianca Luna-Lupercio, Jessica M. Capaldi, Galen Wiens-Cook, Vinicius Calsavara, Zulfikarali Surani, Sarah-Jeanne Salvy, Jane C. Figueiredo, Robert Haile, Nenette A. Cáceres, Celina H. Shirazipour

**Affiliations:** 1 Cedars-Sinai Cancer, Cedars-Sinai Medical Center, 700 N. San Vicente Blvd, G500 West Hollywood, Los Angeles, CA USA; 2 David Geffen School of Medicine, University of California los Angeles, Los Angeles, CA USA

**Keywords:** Colorectal Cancer, Colonoscopy, Latino, Social Stigma, Social Perception

## Abstract

Cancer is a leading cause of death among Hispanic people in the USA. One potential reason is low adherence to cancer screening guidelines, particularly for colorectal cancer (CRC). Previous research suggests that low CRC screening may be linked to negative stereotypes associated with cancer and CRC screening methods. The purpose of this study was to examine explicit stereotype perceptions of CRC screening and diagnosis in the Hispanic community. Hispanic adults (*n* = 279) were asked to read three vignettes presenting a gender-neutral individual who was either diagnosed with CRC, completed colonoscopies for CRC screening, or was a control (no cancer information provided). Using the Stereotype Content Model approach, after reading each vignette, participants completed measures assessing explicit perceptions (warmth and competence) of the individual. Linear mixed-effects models were fitted to evaluate differences in perceptions between vignettes. The main effect indicated no significant difference in warmth or competence perceptions based on vignette condition (*p* = .78). However, there was a significant difference in explicit perceptions based on the study participant’s Hispanic heritage, gender, and age (*p*s < .05). Findings emphasize differences in explicit perceptions of cancer and cancer screening based on important demographic characteristics. Thus, important implications include the need for cancer educational materials and interventions to consider the important heterogeneity within the Hispanic community. Future CRC screening interventions should be tailored based on Hispanic heritage, gender, and age.

## Introduction

Cancer management is often more feasible with early detection [1]. However, cancer screening remains lower in the Hispanic community when compared to non-Hispanic White individuals (NHW) [2]. This is also true in relation to colorectal cancer (CRC). Among Hispanic adults, only 49% of those over the age of 45, and 59% of those over the age of 50, report having a fecal occult blood test in the last year, sigmoidoscopy in the last 5 years, or colonoscopy in the last 10 years, compared to 58% and 68% of NHW individuals [2]. Lower screening rates may be a contributing factor to CRC incidence in the Hispanic population not declining and remaining relatively stable compared to NHW adults [2].

Qualitative research has identified negative perceptions of CRC screening among Hispanic people, with screening methods often viewed as unpleasant and embarrassing [3]. Further qualitative research has suggested that attitudes around fatalism, sexuality, and faith influence perceptions of cancer and can be barriers to cancer screening [3–6]. In the context of cancer, *fatalismo* is the belief that cancer is inevitable and that death is certain once a diagnosis of cancer occurs [7]. Additionally, expressions of *machismo* may hinder screening as colonoscopies can be viewed as stigmatizing among Hispanic men [8]. Specifically, the idea of having a colonoscopy can be perceived as reducing one’s masculinity or even potentially “causing” homosexuality [9]. Faith can also play an important role, particularly the belief that a higher power plays a central role in a patient’s cancer trajectory [10–12].

In light of this research suggesting that stigma — driven by these many factors — may be a reason for lower colorectal cancer screening in the Hispanic community, the current paper seeks to ground our research within a framework of explicit stereotype and stigma perceptions, the Stereotype Content Model (SCM) [13]. The SCM suggests that explicit stigma and stereotypes result from how people perceive individuals on two dimensions: warmth and competence [13]. Different communities (based on race, ethnicity, religion, socioeconomic status, or medical condition) can be mapped according to these high/low warmth and competence dimensions [14]. Based on these evaluations, individuals form emotional prejudices toward the individual they are perceiving. For example, groups perceived as high in warmth but low in competence (e.g., older adults) are approached with pity, whereas groups perceived as high in warmth and competence (e.g., middle-class individuals) are viewed with admiration [13, 15]. These explicit perceptions can promote or inhibit helpful or harmful behaviors and attitudes. For example, pity can result in overtly helpful behaviors and paternalistic attitudes that can lead to humiliation, as well as larger consequences such as unemployment, lower income, and isolation [16, 17]. Certain behaviors, however, such as health behaviors, can mitigate these negative perceptions and outcomes, resulting in admiration and increased social opportunities [13, 15, 18, 19].

Early research suggests that these warmth and competence dimensions may also be influential in relation to how individuals respond to cancer screening and diagnosis [20, 21]. To our knowledge, no studies have used a validated framework, such as the SCM, to quantitatively assess explicit perceptions of CRC screening and diagnosis within the Hispanic community. The aim of this study is to quantitatively examine the Hispanic community’s explicit perceptions of CRC screening and diagnosis using SCM [15]. Based on previous research, we hypothesize that a Hispanic individual diagnosed with CRC will be perceived as high in warmth and low in competence, which would reflect *fatalismo* and pity-based emotions [7]. Meanwhile, a Hispanic individual engaging in CRC screening via colonoscopy would be rated as low warmth and low competence, indicative of contempt-based stereotypes related to stigma towards the cancer screening method [9]. Finally, the control condition, for which no cancer information is provided will receive high warmth and high competence ratings reflective of being seen as a member of the Hispanic community without any contrary characteristics, leading to in-group identification. Using the SCM as a validated framework to examine explicit stereotypes and attitudes toward cancer diagnosis and screening in the Hispanic community will inform the development of effective, culturally sensitive materials and interventions on cancer education, prevention, and survivorship.

## Methods

### Participants

Participant inclusion criteria were (a) English- or Spanish-speaking adults (age ≥ 18); (b) self-identification as Hispanic, Latino, Latina, or Latinx; and (c) lived in the USA.

### Study Design

Ethics approval was obtained from the *removed for anonymous review* institutional ethics board. Participants were recruited through social media advertisements and partnerships with Hispanic community organizations across the USA. Participants could complete the virtual survey in either English or Spanish depending on their language preference.

The study design followed the standard design previously validated for assessing differences in warmth and competence based on a health characteristic or behavior [19, 22–24]. The survey presented three vignettes (control, CRC screening, and CRC diagnosis) to the participants. All were presented in counterbalanced order to minimize order bias. After reading each vignette, participants were asked to answer questions to determine reading comprehension. They were then asked to rate *Alex* using validated warmth and competence surveys [19, 25, 26]. Each vignette and its SCM survey were separated by other surveys including demographics to provide a break between the vignettes.

### Measures

#### Demographics

Participants’ age, sex, gender identity, racial and ethnic background, heritage, place of birth, education status, and cancer exposure were collected.

#### Vignettes

Three vignettes (control, CRC screening, and presence of CRC) were used to represent different conditions. The vignettes were modelled on previous research [19, 22–24] but adapted for cultural sensitivity following discussion with members of a Hispanic research community engagement board. Modeling previous SCM research, the three vignettes were identical except for one sentence, which was manipulated to either note a CRC diagnosis, CRC screening, or no extra sentence (control). The control vignette was presented as follows:

Alex is 53 years old and married with three children. Alex currently lives in the Los Angeles area and is employed full-time. Alex is of average height and weight and has brown eyes and brown hair. When they are not working, Alex enjoys spending time with family. Alex also enjoys listening to music and watching soccer on TV. Alex has three siblings. Alex’s parents and siblings all live nearby. The whole family meets up regularly for family barbecues.

The vignettes portraying the other conditions were the same as above; however, after the sentence describing physical characteristics was either a CRC condition sentence (“Alex hasbeen diagnosed with colorectal cancer”) or a CRC screening condition sentence (“Alex regularly undergoes cancer screening and has a colonoscopy scheduled in a week to screen for colorectal cancer”). The name *Alex* was chosen for the individual described in the vignette after an initial pre-study pilot survey that identified the name as a gender-neutral Hispanic name that could reduce the incidence of gender bias.

#### Warmth and Competence Survey

Competence was assessed across six items (competence, capability, self-confidence, intelligence, efficiency, and skill) and warmth was assessed across six items (warmth, friendliness, well-intentioned, trustworthiness, good-natured, sincerity) [13, 15]. All items were measured on a scale anchored at 1 (*not at all*) and 5 (*extremely*). Validated Spanish translations were used for the Spanish survey [27].

#### Reading Comprehension

After each vignette, participants completed two questions to assess that the vignette had been read and understood. If there were errors, participant surveys were excluded.

### Data Analysis

Competence and warmth scores were calculated by obtaining the average for each vignette. Descriptive statistics were calculated as absolute and relative frequencies for qualitative variables and as means with standard deviations, medians with interquartile ranges for quantitative variables. Bivariate correlations were performed for each condition. A MANOVA was conducted with average warmth and average competence as outcomes and condition as an explanatory factor. Linear mixed-effects models were fitted to evaluate the effect of the condition (control, screening, cancer) in each outcome, warmth, and competence, controlling for age, gender, race, heritage, cancer exposure, and randomization. The models were fitted using the restricted maximum likelihood (REML) estimation considering a random patient effect to describe the measures for the same participant. Interaction between conditions with age, gender, heritage, education, and randomization were evaluated. Satterthwaite’s method was applied for evaluating the significance of fixed effects. Model assumptions were verified using the Shapiro–Wilk test for normality of residuals. Mixed-effects models were fitted using the package lme4 and lmerTest. All hypotheses were two-tailed with a 5% significance level.

## Results

A total of 279 adults (*M*_age_, 33.94 ± 8.81) participated. Full demographic characteristics are provided in Table 1.

**Table 1:** Demographic characteristics

Demographic characteristics	*M* (SD)/*n* (%)
Age	33.95 (8.81)
Sex assigned at birth	
Male	177 (64.33)
Female	100 (35.84)
Decline to answer	2 (0.72)
Gender	
Cisgender man	170 (60.93)
Cisgender woman	106 (37.99)
Transgender woman	3 (1.08)
Race	
Hispanic White	250 (89.61)
Black or African American	5 (1.79)
Multiracial	7 (2.15)
Decline to answer	17 (6.09)
Hispanic heritage	
South American	87 (31.18)
Mexican	61 (21.86)
European	58 (20.79)
Caribbean	25 (8.96)
Mixed	25 (8.96)
Central American	19 (6.81)
Decline to answer	4 (1.43)
Education	
Bachelors	132 (47.31)
High school or GED	67 (24.01)
Vocational or associate’s degree	49 (17.56)
Graduate degree	24 (8.60)
Junior high or less	6 (2.15)
Decline to answer	1 (0.36)
Cancer exposure	
Knows someone diagnosed	143 (52.38)
Neither diagnosed nor knows someone diagnosed	67 (24.01)
Diagnosed themselves	13 (4.67)
Diagnosed and knows someone	8 (2.94)

Pearson correlations between warmth and competence were large across conditions: 0.73 (95% CI, 0.67 – 0.78) for cancer, 0.76 (95% CI, 0.70 – 0.80) for control, and 0.82 (95% CI, 0.77 – 0.85) for screening (*p*s < 0.001). An unadjusted MANOVA model did not reveal significant differences between warmth and competence on the different conditions (Wilks’ Lambda *p* = 0.78). Linear mixed models indicated that the target condition(s) of a CRC diagnosis or CRC screening was not statistically significantly associated with warmth and competence when the models were fitted considering only the main effects. Means and standard deviations for competence and warmth across conditions are presented in Table 2.

**Table 2:** Mean and standard deviation of warmth and competence by target condition

Outcome	Control	Cancer screening	Cancer diagnosis
	*M* (SD)	*M* (SD)	*M* (SD)
Competence	3.44 (0.70)	3.48 (0.72)	3.46 (0.66)
Warmth	3.49 (0.71)	3.56 (0.71)	3.53 (0.72)

*M* mean, *SD* standard deviation

While there was no statistically significant differences considering only the main effects, there were significant differences based on three demographic variables: heritage, gender, and age.

### Heritage

#### Warmth

The analysis yielded a significant difference between the average warmth scores for all target conditions and heritage [*F*(8, 520.16) = 2.299, *p* = 0.020] such that those with Mexican heritage rated targets as significantly higher in warmth compared to those with Central American, South American, and European heritage (*p*s < 0.0001). Specific to the CRC screening target, those with mixed heritage and those who did not report heritage rated the targets as significantly lower in warmth compared to those with Mexican heritage (*p*s ≤ 0.0001).

#### Competence

Similar to warmth, there was a significant difference in competence based on heritage [*F*(4, 248.30) = 3.530, *p* = 0.008]. Participants with Mexican heritage rated the targets as having higher average competence across all conditions compared to those with Central American (*p* = 0.001), South American (*p* = 0.001), and European heritage (*p* = 0.002).

#### Interaction

The interaction term in a regression of competence and warmth on heritage by condition did not show significance (*p*s > 0.05).

### Gender

#### Warmth

The analysis also yielded a significant difference in warmth of the targets based on gender [(*F*(2, 239.03) = 3.119, *p* = 0.046]. Across all conditions, men rated participants lower in warmth compared to women (*p* = 0.015).

#### Competence

There was a significant difference in perceptions of competence across all conditions based on participant gender [*F*(2, 240.38) = 3.282, *p* = 0.039]. Men rated participants lower in competence across all conditions compared to women (*p* = 0.012).

#### Interaction

The interaction term in a regression of competence and warmth on gender by condition did not show significance (*p*s > 0.05).

### Age

#### Warmth

Differences were identified for the cancer screening target, with younger participants rating the target higher in warmth compared to older participants (*p* ≤ 0.0001).

#### Competence

As with the warmth outcome, younger participants rated the cancer screening target higher in competence compared to older participants (*p* < 0.0001).

#### Interaction

**Fig. 1:**
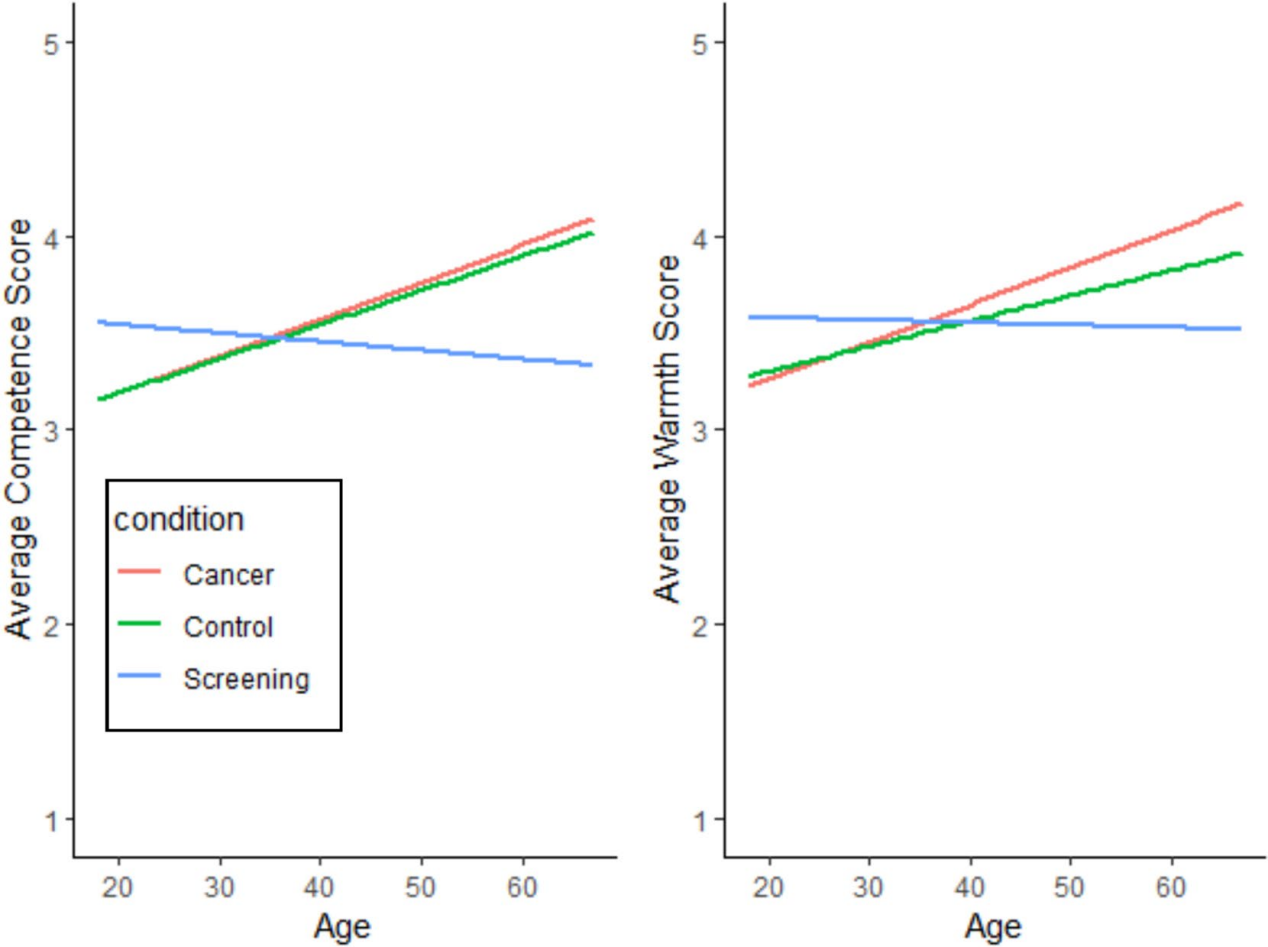
Interaction of target condition by age

The analysis yielded a significant fixed effect for the interaction between target condition and participant age, such that older participants had greater perceptions of warmth and competence towards the CRC diagnosis and control targets but not the target undergoing cancer screening (see Fig. 1 and Table 3).

**Table 3:** Interaction of target condition by age

Outcome	Target condition	Slope	SE	*p*-value
Competence				
	CRC diagnosis	.018	.004	< .0001
	CRC screening	− .004	.005	.371
	Control	.019	.005	.0002
Warmth				
	CRC diagnosis	.019	.005	< .0001
	CRC screening	− .001	.005	.796
	Control	.013	.005	< .0001

Interaction tests for whether there was a difference among the three slopes significant for competence (*p* = .0004) and warmth (*p* = .0087)

*CRC* colorectal cancer

## Discussion

These findings have important implications for the precision tailoring of CRC educational materials and screening interventions in the Hispanic community. Our hypothesis that the CRC target or the target undergoing CRC screening would be rated lower in warmth and competence than the control was not upheld. Further analysis did, however, identify significant differences based on three important demographic characteristics: age, heritage, and gender.

First, there was an interaction between older age and the CRC target, such that older participants rated the CRC diagnosis target higher in warmth and competence. Familiarity with cancer in older age through individual, friend, or family diagnoses may lead to greater association with the cancer diagnosis target. Conversely, younger Hispanic participants rated the target undergoing CRC screening with higher warmth and competence. Per the SCM, this suggests that younger members of the Hispanic community rate CRC screening behaviors as more admirable and less stigmatizing [13]. Previous qualitative research explored the influences of *machismo* and *fatalismo* among adults with a mean age of 56 [8], which may explain the lower ratings for the screening condition among older adults. Future research should seek to better understand the generational nature of these values in relation to cancer screening, particularly as younger age is associated with a higher likelihood of CRC screening among Hispanic individuals [28].

Second, those with Mexican heritage rated all conditions with higher warmth and competence compared to those with Central American, South American, and European Hispanic heritage. Nativity status has previously been found to predict cancer screening within the Hispanic community [3]. Differences in access to health services and understanding of cancer and screening based on place of origin may have influenced participants’ perceptions toward the screening target [29, 30]. Findings reinforce the need for future research exploring the diversity in screening perceptions and behaviors based on heritage, as well as the importance of Hispanic heritage as a consideration in clinical settings.

The final demographic characteristic that influenced perceptions was gender. While the overall ratings were not stigmatizing, they do suggest emotional prejudice may differ between Hispanic men and women. More negative perceptions of the cancer targets among Hispanic men aligns with existing qualitative research that identified colonoscopy-related stigma [8]. In contrast, women perceived the cancer targets with less emotional prejudice, rating the target with higher warmth and competence. Women’s perceptions may be influenced by gender-specific beliefs that facilitate more proactive health behaviors. For example, Hispanic women are more likely to have undergone a colonoscopy than men [31]. Gender-specific cultural norms may facilitate health behaviors that align with the cancer targets in the vignettes, thus resulting in more favorable perceptions among Hispanic women compared to men.

This study had a number of strengths, including diverse representation of the Hispanic community and the use of an evidence-based framework to examine explicit perceptions of CRC. However, limitations need to be considered. Most notably, rooted within SCM, our focus was on explicit perceptions. This may account for the differences in findings compared to other research, as implicit perceptions may also inform how participants perceived the target. Future research can assess implicit perceptions to CRC screening interventions and materials through physical reactions such as eye tracking, galvanic skin response, pupil size, and body language. Second, in alignment with previous qualitative research, the focus of the vignettes was on colonoscopy screening. However, different types of screenings exist for CRC, including stool tests. Future research should explore the diverse stereotypes and biases surrounding these different screening methods. Third, our findings indicated the importance of age in explicit perceptions. This study included participants ages 18 and older. However, CRC screening guidelines only recommend screening for ages 45 to 75. Thus, many of the younger study participants may not have experienced CRC screening decision-making or CRC screening, which can influence their explicit perceptions and ability to accurately rate the vignettes. Finally, future research should also assess factors qualitative research has indicated as influencing perceptions, including machismo and fatalismo. As an initial examination of the topic of perceptions of CRC and CRC screening in the Hispanic community, the current analysis focused on demographics in relation to explicit perceptions. However, future analyses should take into consideration the impact of how an individual identifies with these cultural perceptions.

In conclusion, findings reinforce the diversity within the Hispanic community and the resulting impact on perceptions of CRC and CRC screening. This has important implications by demonstrating the need for screening and survivorship educational materials and interventions to respect the diversity and richness of the Hispanic community and reflect the heterogeneity and layered richness of perceptions.

## Data Availability

Data is available from the corresponding author upon request.

## References

[1] Miller KD et al (Nov2021) Cancer statistics for the US Hispanic/Latino population, 2021, (in eng). CA Cancer J Clin 71(6):466–487. 10.3322/caac.21695 34545941 10.3322/caac.21695

[2] Society AC. Cancer facts & figures for Hispanics/Latinos 2021–2023, (in en). [Online]. Available: https://www.cancer.org/content/dam/cancer-org/research/cancer-facts-and-statistics/cancer-facts-and-figures-for-hispanics-and-latinos/hispanic-latino-2021-2023-cancer-facts-and-figures.pdf

[3] Walsh JM, Kaplan CP, Nguyen B, Gildengorin G, McPhee SJ, Pérez-Stable EJ (2004) Barriers to colorectal cancer screening in Latino and Vietnamese Americans. Compared with non-Latino white Americans, (in eng). J Gen Intern Med 19(2):156–66. 10.1111/j.1525-1497.2004.30263.x 10.1111/j.1525-1497.2004.30263.x PMC1492137 15009795

[4] Abraído-Lanza AE, Viladrich A, Flórez KR, Céspedes A, Aguirre AN, De La Cruz AA (2007) Commentary: fatalismo reconsidered: a cautionary note for health-related research and practice with Latino populations, (in eng). Ethn Dis 17(1):153–8 PMC3617551 17274225

[5] Leyva B, Allen JD, Tom LS, Ospino H, Torres MI, Abraido-Lanza AF (2014) Religion, fatalism, and cancer control: a qualitative study among Hispanic Catholics, (in eng). Am J Health Behav 38(6):839–849. 10.5993/ajhb.38.6.6 25207510 10.5993/AJHB.38.6.6 PMC4424042

[6] Ramírez AS et al (2013) Perceptions of cancer controllability and cancer risk knowledge: the moderating role of race, ethnicity, and acculturation, (in eng). J Cancer Educ 28(2):254–261. 10.1007/s13187-013-0450-8 23355279 10.1007/s13187-013-0450-8 PMC4758124

[7] Allen JD et al (2014) Religious beliefs and cancer screening behaviors among Catholic Latinos: implications for faith-based interventions, (in eng). J Health Care Poor Underserved 25(2):503–526. 10.1353/hpu.2014.0080 24858865 10.1353/hpu.2014.0080 PMC4162660

[8] Powe BD, Finnie R (2003) Cancer fatalism: the state of the science, (in eng). Cancer Nurs 26(6):454–65. quiz 466–7. 10.1097/00002820-200312000-00005 10.1097/00002820-200312000-00005 15022977

[9] Mojica CM, Vargas N, Bradley S, Parra-Medina D (2023) Barriers and facilitators of colonoscopy screening among Latino men in a colorectal cancer screening promotion program, (in eng). Am J Mens Health 17(3):15579883231179325. 10.1177/15579883231179325 10.1177/15579883231179325 PMC10262627 37287187

[10] Getrich CM et al (Apr2012) Expressions of machismo in colorectal cancer screening among New Mexico Hispanic subpopulations, (in eng). Qual Health Res 22(4):546–559. 10.1177/1049732311424509 22138258 10.1177/1049732311424509 PMC3636712

[11] Sommariva S et al (2019) Hispanic male cancer survivors’ coping strategies. Hisp J Behav Sci 41(2):267–284. 10.1177/0739986319840658

[12] Carrion IV, Nedjat-Haiem F, Macip-Billbe M, Black R (2017) “I told myself to stay positive” perceptions of coping among Latinos with a cancer diagnosis living in the United States, (in eng). Am J Hosp Palliat Care 34(3):233–240. 10.1177/1049909115625955 26764346 10.1177/1049909115625955

[13] Larson KL et al (2021) Four kinds of hard: an understanding of cancer and death among Latino community leaders, (in eng). Glob Qual Nurs Res 8:23333936211003557. 10.1177/23333936211003557 10.1177/23333936211003557 PMC7992742 33816705

[14] Cuddy AJC, Fiske ST, Glick P (2007) The BIAS map: behaviors from intergroup affect and stereotypes, (in eng). J Pers Soc Psychol 92(4):631–648. 10.1037/0022-3514.92.4.631. 10.1037/0022-3514.92.4.631 17469949

[15] Cuddy AJC, Fiske ST, Glick P (2008) Warmth and competence as universal dimensions of social perception: the stereotype content model and the BIAS map, in. Adv Exp Soc Psychol 40: Academic Press. pp. 61–149

[16] Fiske ST, Cuddy AJC, Glick P, Xu J (2002) A model of (often mixed) stereotype content: competence and warmth respectively follow from perceived status and competition. J Pers Soc Psychol 82(6):878–902. 10.1037/0022-3514.82.6.878 12051578

[17] Hogan A, Kyaw-Myint SM, Harris D, Denronden H (2012) Workforce participation barriers for people with disability. Int J Disabil Manag 7:1–9. 10.1017/idm.2012.1

[18] Macdonald SJ et al (2018) ‘The invisible enemy’: disability, loneliness and isolation. Disabil Soc 33(7):1138–1159. 10.1080/09687599.2018.1476224

[19] Lindwall M, Martin Ginis KA (2006) Moving towards a favorable image: the self-presentational benefits of exercise and physical activity. Scand J Psychol 47(3):209–217. 10.1111/j.1467-9450.2006.00509.x 10.1111/j.1467-9450.2006.00509.x 16696845

[20] Gainforth H, O’Malley D, Mountenay T, Latimer A (2013) Independence and physical activity status moderate stereotypes toward people with a physical disability. Int J Sport Exerc Psychol 11. 10.1080/1612197X.2013.749001

[21] Clément-Guillotin C, Falzon C, d’Arripe-Longueville F (2015) Can exercise change the stereotypes associated with individuals with cancer? Scand J Med Sci Sports 25(4):552–557. 10.1111/sms.12272 24979050 10.1111/sms.12272

[22] Martinez L, White C, Shapiro J, Hebl M (2015) Selection BIAS: stereotypes and discrimination related to having a history of cancer. J Applied Psychol 101. 10.1037/apl0000036 10.1037/apl0000036 26121089

[23] Arbour KP, Latimer AE, Ginis KAM, Jung ME (2007) Moving beyond the stigma: the impression formation benefits of exercise for individuals with a physical disability. Adapt Phys Activ Q 24(2):144–159 17916914 10.1123/apaq.24.2.144

[24] Ginis KAM, Latimer AE, Jung ME (2003) No pain no gain? Examining the generalizability of the exerciser stereotype to moderately active and excessively active targets, Social Behavior and Personality: an international journal 31(3):283–290

[25] Shirazipour CH, Munroe-Chandler KJ, Loughead TM (2017) Judging the gym: stereotypes of female weight trainers. Int J Sport Exerc Psychol 15(4):337–350. 10.1080/1612197X.2016.1153128

[26] Shirazipour CH, Stone RC, Lithopoulos A, Capaldi JM, Latimer-Cheung AE (2023) Examining the impact of the Rio 2016 paralympic games on explicit perceptions of paralympians and individuals with disabilities. Health Commun 38(8):1501–1507. 10.1080/10410236.2021.2017107 10.1080/10410236.2021.2017107 34984933

[27] Dionne CD, Gainforth HL, O’Malley DA, Latimer-Cheung AE (2013) Examining implicit attitudes towards exercisers with a physical disability, (in eng). Sci World J 2013:621596. 10.1155/2013/621596 10.1155/2013/621596 PMC3654286 23710142

[28] Nicolas G, Bai X, Fiske ST (2021) Comprehensive stereotype content dictionaries using a semi-automated method. Eur J Soc Psychol 51(1):178–196

[29] Espinoza-Gutarra MR et al (Mar2023) Cancer-related knowledge, beliefs, and behaviors among Hispanic/Latino residents of Indiana, (in eng). Cancer Med 12(6):7470–7484. 10.1002/cam4.5466 36683200 10.1002/cam4.5466 PMC10067073

[30] Rosal MC, Wang ML, Silfee VJ (2023) Culture, behavior, and health. Springer Publishing Company. https://connect.springerpub.com/content/book/978-0-8261-8014-8/part/part02/chapter/ch05

[31] Kagawa Singer M (2012) Applying the concept of culture to reduce health disparities through health behavior research. Prev Med 55(5):356–361. 10.1016/j.ypmed.2012.02.011 10.1016/j.ypmed.2012.02.011 22391576

